# A Compact Vertical Scanner for Atomic Force Microscopes

**DOI:** 10.3390/s101210673

**Published:** 2010-11-30

**Authors:** Jae Hong Park, Jaesool Shim, Dong-Yeon Lee

**Affiliations:** 1 NEMS and Bio Team, National Nano-fab Center, 335, Gwahangno, Yuseong-gu, Daejeon-si, 305-806, Korea; E-Mail: jhpark@nnfc.re.kr; 2 School of Mechanical Engineering, Yeungnam University, 214-1, Dae-dong, Gyeongsan-si, Gyeongsangbukdo, 712-749, Korea; E-Mail: jshim@ynu.ac.kr

**Keywords:** nano-scanner, atomic force microscope, flexure-guide, piezoelectric actuator, nano-sensor

## Abstract

A compact vertical scanner for an atomic force microscope (AFM) is developed. The vertical scanner is designed to have no interference with the optical microscope for viewing the cantilever. The theoretical stiffness and resonance of the scanner are derived and verified via finite element analysis. An optimal design process that maximizes the resonance frequency is performed. To evaluate the scanner’s performance, experiments are performed to evaluate the travel range, resonance frequency, and feedback noise level. In addition, an AFM image using the proposed vertical scanner is generated.

## 1. Introduction

An atomic force microscope (AFM) is composed of a micro-machined cantilever with nulling control devices, vertical and horizontal scanners (usually, monolithic tube piezoelectric scanners), an optical microscope for viewing the cantilever and samples, and coarse positioning devices for the cantilever and the sample [1]. The optical microscopes are configured into two types. One type is on-axis, wherein the optical center axis of the optical microscope coincides with the cantilever’s axis. The other type is off-axis, wherein the optical axis of the optical microscope doesn’t coincide with the cantilever’s axis. Because the viewing angle of the on-axis optical microscope is limited, the AFM head, including the vertical scanner, must not interfere with the viewing angle for clear imaging of the cantilever and sample. In addition, previous studies have mainly considered horizontal *xy*-scanners [2–5] or the coupled xyz-scanner [6]. Recently, the compact two-axis scanner with scan range of 10 μm × 10 μm was developed for high speed scanning [7].

We have developed a compact vertical scanner that has no interference with the viewing angle of the optical microscope. The scanner is composed of a linear flexure guide, a piezoelectric actuator, and a feedback sensor. The theoretical stiffness of the flexure guide is analyzed, and its resonance is calculated and verified using finite element analysis. The optimal design technique is used to maximize the feedback speed, and the result is verified using finite element analysis. The travel range, feedback noise, nulling resolution, and resonances were evaluated experimentally and compared to the theoretical findings. Finally, we present an AFM image from the developed vertical scanner.

## 2. Optimal Design of the Vertical Scanner

**Figure 1.**
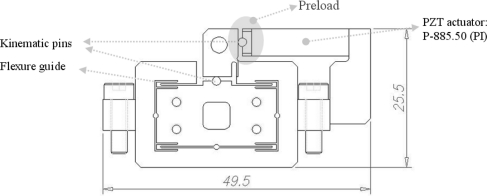
Schematic diagram of the *z*-scanner.

A *z*-scanner serves as a vertical fine scanner for the nulling control of an optical lever. A diagram of the *z*-scanner is shown in Figure 1. The flexure guide is used as a guide mechanism. A PZT actuator is attached to the flexure guide via a kinematic pin. Because the PZT actuator is fragile with respect to tension and moment loading, it cannot be glued into the guide. As a solution, an appropriate preload is applied to the flexure guide during assembly to prevent detachment between the actuator and the guide. Accordingly, the preloading effect should be considered in the optimal design. The critical design point of the scanner is as follows. Because the working distance and the viewing angle of the objective lens are fixed, the height and the overall dimension of the *z*-scanner should be minimized for viewing the cantilever. In this study, so as not to interfere with the viewing angle of the objective lens, the PZT actuator’s horizontal deformations are transferred to the vertical flexure guide via the circular hinge and the two kinematic pins. Moreover, the resonant frequency should be high enough for fast nulling control.

**Figure 2.**
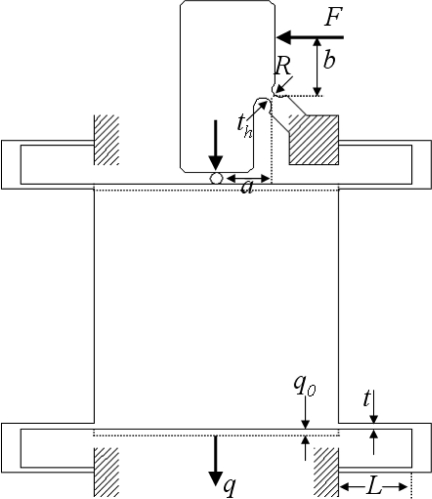
A simplified model of the *z*-scanner.

For the model, the *z*-scanner is simplified as shown in Figure 2. *F* is a force exerted by the PZT actuator; *R*, *t_h_*, and *w_h_* are the radius, thickness, and depth (width) of the rotational hinge, respectively; *L*, *t*, and *w* are the length, thickness, and depth (width) of the flexure guide; and *q* and *q_0_* are displacement and initial preload of the moving body, respectively.

The static stiffness of the flexure guide is given by [8]:

(1) $$k_{f}=\frac{24EI}{L^{3}}$$

where *E* is Young’s modulus and *I* (= *wt*^3^/12) is the second moment of inertia of the flexure guide. The stiffness (N/m) of the rotational hinge for changing of the force exerted by the PZT actuator is calculated as follows:

(2) $$k_{h}=\frac{{2Ew}_{h}t^{2.5}}{9\pi R^{0.5}ab}$$

Therefore, the total stiffness of the *z*-scanner is given by:

(3) $$k_{t}=k_{f}+k_{h}=\frac{24EI}{L^{3}}+\frac{{2Ew}_{h}t^{2.5}}{9\pi R^{0.5}ab}$$

Using Equation (3) and considering the PZT specifications, the displacement of the flexure guide due to the PZT force is given by:

(4) $$q=\frac{F_{\text{max}}d_{\text{max}}}{F_{\text{max}}+d_{\text{max}}k_{t}}-\frac{d_{\text{max}}k_{t}q_{0}}{F_{\text{max}}+d_{\text{max}}k_{t}}$$

where *F_max_* and *d_max_* are the maximum force and the maximum displacement of the PZT actuator at the full voltage input. As described in Equation (4), the initial preload *q_0_* decreases the total displacement.

Using Equation (3), the natural frequency of the flexure guide is calculated as follows:

(5) $$f_{n}=\frac{1}{2\pi }\sqrt{k_{f}/m_{f}}$$

where *m_f_* is the moving mass of the flexure guide.

The optimal design is determined with the objective of maximizing the resonant frequency. Thus, the cost function is given by:

(6) $$J=\text{min}\left(\frac{1}{f_{s}^{2}}\right)$$

The cost function has two constraints: the maximum displacement should be larger than 17 μm, and the maximum stress should be lower than 20% of the ultimate strength of the material. The maximum stress was selected considering the fatigue fracture based on the following equation:

(7) $$S_{n}=ɛC_{L}C_{G}C_{S}S_{\text{nn}}$$

where *S_n_* is the actual endurance limit after 10^8^ cycles, *ɛ* is the uncertainty (0.92) in the endurance limit, *C_L_* is the load factor (1 for bending), *C_G_* is the gradient factor (0.9), *C_S_* is the surface factor (0.9 for a commercially polished surface), and *S_nn_* is the endurance limit of the ideal material (0.35 × *S_u_* (ultimate strength)). Therefore, *S_n_* = 0.26 × *S_u_*. The maximum stress was selected to be lower than 20% of the ultimate strength of the material, thereby allowing for some margin. The constraint equations are summarized as follows:

(8) $$\begin{array}{l} q_{\text{max}}>17\;\mu m\\ \sigma_{\text{max}}<0.2(S_{u}) \end{array}$$

The optimal parameters were selected as shown in Table 1.

**Figure 3.**
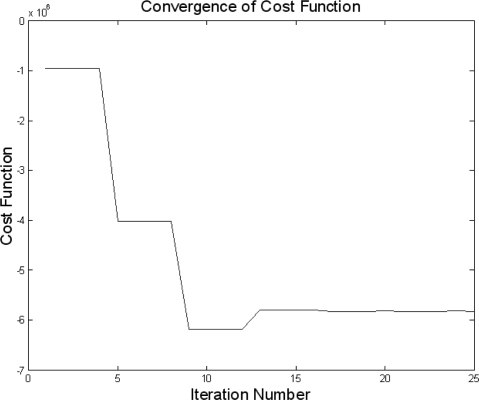
Convergence plot of the optimization process.

Using the sequential quadratic programming (SQP) method of MATLAB^®^ Optimization Toolbox™ (The MathWorks), the convergence plot after completion of the optimization process was determined as shown in Figure 3.

**Figure 4.**
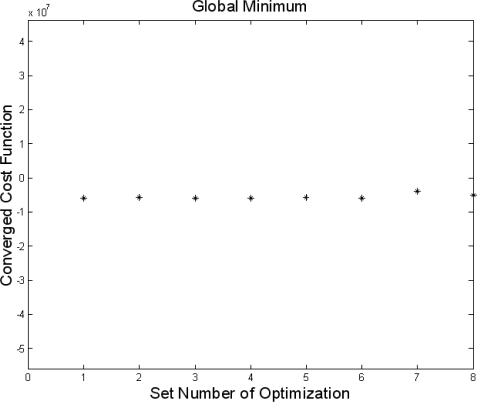
Convergence of the cost function at eight different initial values. This plot shows that the optimized results are global minimums.

To ensure a global minimum value, eight optimization processes with random initial values were performed as shown in Figure 4. The optimized values are summarized as shown in Table 2.

**Figure 5.**
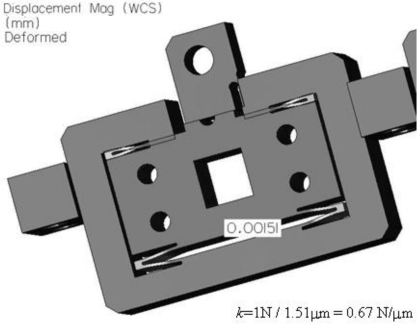
Static FEA of the *z*-scanner.

**Figure 6.**
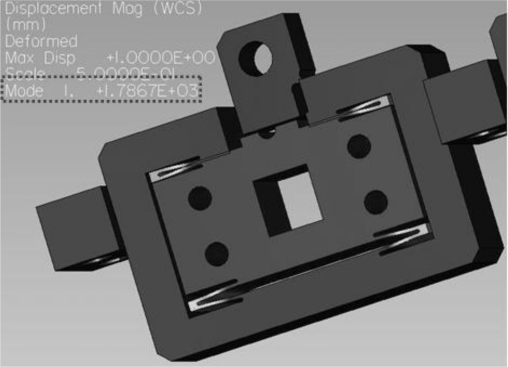
Dynamic FEA of the *z*-scanner.

To validate the optimal design, finite element analyses using the commercial program Pro/ENGINEER Mechanica™ were performed, and the results are shown in Figure 5 and Figure 6. The stiffness simulation result is shown in Figure 5 and the first resonant frequency is shown in Figure 6. The comparison between the optimal design and the FEA results are summarized in Table 3. The difference is within 10%, which is acceptable. If the total moving mass, including the optical lever, is considered in the calculation of the resonant frequency, the final resonant frequency is 2.1 kHz.

## 3. Experiments

**Figure 7.**
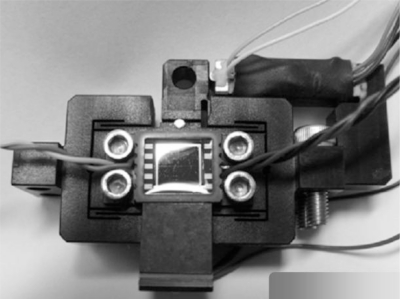
Photo of the *s*-scanner inside of the head.

**Figure 8.**
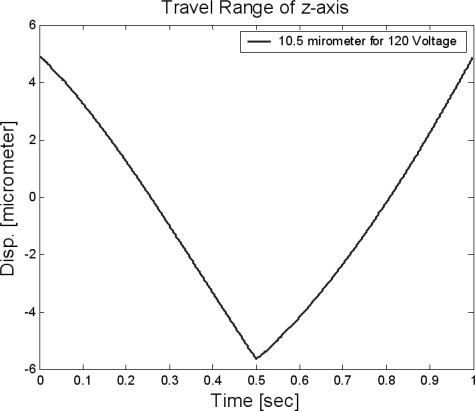
Travel range of the *z*-scanner.

**Figure 9.**
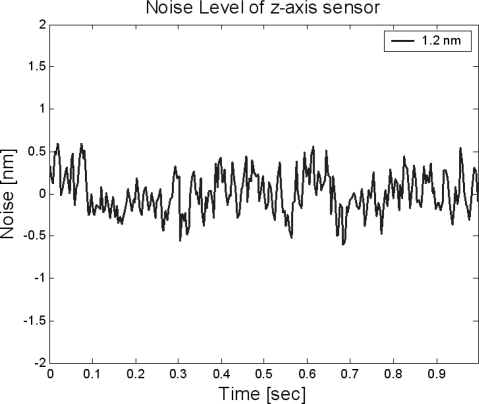
Noise level of the *z*-scanner.

**Figure 10.**
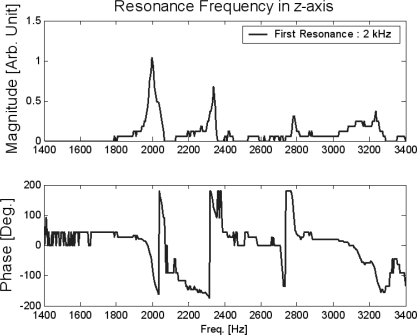
Resonances of the *z*-scanner.

The assembled *z*-scanner is shown in Figure 7. The travel range of the *z*-scanner is shown in Figure 8. Here, the range is about 10.5 μm. The input voltage is about 120 V for protection of the PZT actuator. If an input voltage of 140 V (which is the maximum input voltage for the PZT actuator) is given, the travel range would increase to 13 μm which is close to the final goal of the design. The resolution was measured as shown in Figure 9. The noise is about 1.2 nm peak-to-peak. Also, the resonance was measured as shown in Figure 10. The first resonance is about 2 kHz, which is close to the optimal design result.

**Figure 11.**
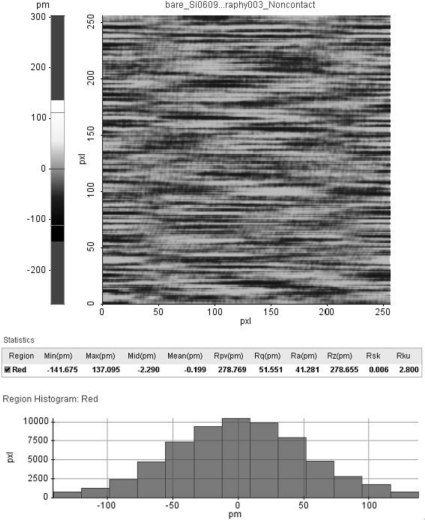
Vertical nulling resolution of the TS-AFM. Top: NC-AFM image. Middle: statistics. Bottom: histogram.

As a first measurement, the vertical nulling resolution of the total system is an important factor that determines the quality of the AFM image. The vertical nulling resolution characterizes the stability of maintaining the gap between the tip and sample. After approaching the tip close to the sample surface, we measured the gap between the tip and sample using the optical lever of the AFM head at the null state of the cantilever with no *xy* scanning. In this situation, a vertical AFM signal is generated by external noise (floor vibration, *etc*.) and internal electronic noise. This vertical AFM signal corresponds to the vertical resolution of the AFM because there is no meaningful atomic force or scanning. Figure 11 shows a histogram of the gap between the tip and sample. The vertical resolution was measured as 0.05 nm root-mean-square (RMS) from Figure 11.

**Figure 12.**
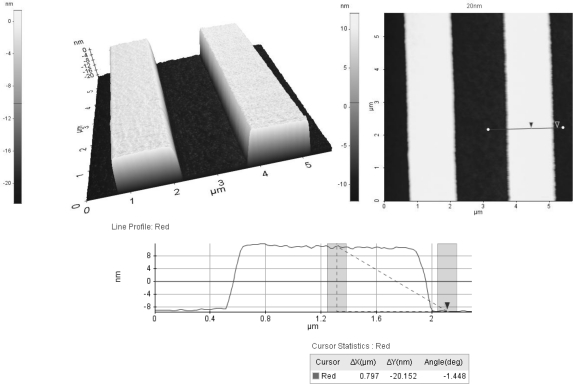
Non-contact AFM image of the 20 nm standard height grating.

The step height (20 nm) standard sample was measured using our homemade AFM with the proposed vertical scanner as shown in Figure 12. The step height sample is clearly shown in the figure, and the vertical scanner was shown to be useful and applicable to the AFM.

## 4. Summary and Conclusions

A compact AFM vertical scanner that has no interference with the viewing angle of the optical microscope was developed. The theoretical stiffness of the flexure guide was analyzed, and the resonance was calculated and verified via finite element analysis. A design optimization process to maximize the feedback speed was performed and verified via finite element analysis. The travel range, feedback noise, nulling resolution, and resonances were experimentally evaluated and compared to the theoretical findings. The travel range was measured as 10.5 μm for a 120 V input. The feedback noise was about 1.2 nm peak-to-peak. The first resonance is about 2 kHz, which is close to the optimal design results. Finally, a non-contact AFM image of the 20 nm height standard sample was generated. As a final comment, the travel range of the scanner is about 10 μm that seems to be large for the specialized wafer industries. Because the usual measuring height is less than 1 μm in the wafer industries, so the small actuator could be used for the above specialized application field. If the nano-scanner is made from the vacuum compatible materials, the nano-scanner could be used in the scanning electron microscope.

## Figures and Tables

**Table 1.** Optimization parameters.

**Item**	**Lower Bound**	**Upper Bound**	**Initial Values**
Preload (*q_0_*, μm)	5	20	Random value within lower and upper bounds.
Thickness (*t*, mm)	0.2	0.5	

Length (*L*, mm)	4	9	

**Table 2.** Optimized results of parameters

	
	**Optimum Values**	**Design Values**
Thickness (mm)	0.32	0.3
Length (mm)	4.8	4.8
Preload (*q_0_*, μm)	20	20
Resonance frequency (kHz)	1.95	

Maximum displacement = 17.4 μm, Maximum stress = 54 MPa		

**Table 3.** Comparison between the optimal and FEA solutions.

	
	**Analytic sol.**	**FEA sol.**	**Error (%)**
Stiffness of the guide (N/μm)	0.71	0.67	5.6
First resonant frequency of the guide (kHz)	1.95	1.78	8.7
Moving mass of *z*-scanner: 4.7 gram			
